# Sun protection and sunbathing practices among at-risk family members of patients with melanoma

**DOI:** 10.1186/1471-2458-11-122

**Published:** 2011-02-21

**Authors:** Sharon L Manne, Elliot J Coups, Paul B Jacobsen, Michael Ming, Carolyn J Heckman, Stuart Lessin

**Affiliations:** 1 Cancer Prevention and Control Program, The Cancer Institute of New Jersey, University of Medicine and Dentistry of New Jersey, New Brunswick, New Jersey, USA; 2 Department of Health Outcomes and Behavior, Moffitt Cancer Center, Tampa, Florida, USA; 3 Department of Dermatology, School of Medicine, University of Pennsylvania, Philadelphia, Pennsylvania, USA; 4 Cancer Prevention and Control Program, Fox Chase Cancer Center, Philadelphia, Pennsylvania, USA; 5 Department of Medicine, Fox Chase Cancer Center, Philadelphia, Pennsylvania, USA

## Abstract

**Background:**

Despite the increased level of familial risk, research indicates that family members of patients with melanoma engage in relatively low levels of sun protection and high levels of sun exposure. The goal of this study was to evaluate a broad range of demographic, medical, psychological, knowledge, and social influence correlates of sun protection and sunbathing practices among first-degree relatives (FDRs) of melanoma patients and to determine if correlates of sun protection and sunbathing were unique.

**Methods:**

We evaluated correlates of sun protection and sunbathing among FDRs of melanoma patients who were at increased disease risk due to low compliance with sun protection and skin surveillance behaviors. Participants (*N *= 545) completed a phone survey.

**Results:**

FDRs who reported higher sun protection had a higher education level, lower benefits of sunbathing, greater sunscreen self-efficacy, greater concerns about photo-aging and greater sun protection norms. FDRs who reported higher sunbathing were younger, more likely to be female, endorsed fewer sunscreen barriers, perceived more benefits of sunbathing, had lower image norms for tanness, and endorsed higher sunbathing norms.

**Conclusion:**

Interventions for family members at risk for melanoma might benefit from improving sun protection self-efficacy, reducing perceived sunbathing benefits, and targeting normative influences to sunbathe.

## Background

Melanoma is the most deadly form of skin cancer, accounting for more than 70% of skin cancer deaths in the United States [1]. The incidence of melanoma is increasing rapidly [2] and faster than any other type of cancer [1]. Family history of melanoma is a known independent risk factor for melanoma [3]. While intense sun exposures and sunburns before the age of 18 are known risk factors, sun exposure during adulthood also impacts melanoma development [4,5]. The American Academy of Dermatology [6], the American Cancer Society [7], the Centers for Disease Control and Prevention [8], and the Task Force on Community Preventive Services on Reducing Exposure to Ultraviolet Light [9] recommend sun avoidance during peak ultraviolet light (UV) hours and use of sun protective clothing for the general population. First degree relatives (FDRs) of individuals who receive a diagnosis of melanoma are at increased disease risk and should pay special attention to precautions to limit sun exposure (e.g., [10]).

Despite the increased level of familial risk, results of several studies indicate that family members of patients with melanoma engage in relatively low levels of UV protection and high levels of exposure. Bergenmaar and Brandberg [11] assessed young adults with a family history of melanoma and found that engagement in sun protection was low and that sun exposure was high. Almost a third of the sample reported sunbathing very often or often and 28% reported using a tanning bed at least once per month in the past year. Geller and colleagues [12] found that about half of the adult siblings of individuals diagnosed with melanoma did not report using sunscreen regularly. Manne and colleagues [13] reported that FDRs of individuals diagnosed with melanoma engaged in relatively low levels of sun protection. Sunbathing was not assessed in this study. Azzarello and colleagues [14] assessed sun protection practices among FDRs of individuals diagnosed with melanoma, and reported that more than one-third of relatives never or rarely used sunscreen, and more than 60% rarely or never wore protective clothing. Again, sunbathing was not assessed. Geller and colleagues [15] studied children of individuals diagnosed with all skin cancer types and found that use of sunscreen was relatively low (42%). Rates of frequent sunburn in the past year were also relatively high (39%), with particularly high rates of sunburn in the past year among female offspring of mothers who had received a diagnosis of skin cancer. Finally, Bishop and colleagues [16] studied sun protection and sun exposure individuals with a first degree relative with melanoma and found that about 33% of relatives had a sunburn in the previous summer and 64% reported getting a tan the previous summer. However, sunscreen use was high in this sample (90%) as was the use of other methods of sun protection.

Several studies have examined correlates of sun protection practices among relatives of individuals diagnosed with melanoma. These studies have focused on demographic, phenotypic, health care access, and attitudinal factors. In terms of demographic factors, some studies suggest that female gender [12] and a college education [14] are associated with greater sun protection, whereas other studies do not suggest these associations [11,13]. In terms of phenotypic factors, a greater tendency to burn [12] and greater number of melanoma risk factors [14] have been associated with sun protection in some studies, but not in others [13]. Health care access and knowledge factors such as having a dermatologist [12], a physician recommendation to engage in sun protection [13], and a greater knowledge level regarding what suspicious moles look like [12] have been associated with higher engagement in sun protection. Attitudinal factors such as a greater perceived risk [14] have been associated with greater sun protection habits in some studies [14] but not others [12,13]. Greater self-efficacy has been consistently associated with engagement in sun protection [13,14]. Fewer perceived barriers to using sunscreen [13] and lower normative influences for sunbathing [11,13] have also been associated with sun protection. Appearance benefits and normative influences have been described as common reasons for sunbathing among relatives [11].

Although there have been several studies focusing on sun habits of family members of melanoma patients, there are two gaps in the literature. First, no study has evaluated the role of a comprehensive set of attitudinal and knowledge factors in ***both ***sun protection and sunbathing practices among family members and compared whether the correlates of each behavior differ. The majority of studies have studied sun protection with little attention paid to correlates of sunbathing. Second, little is known about the population of relatives who are the least compliant with skin protection behaviors. This is a little-studied population that is most reluctant to adopt sun protection. It is important to better understand their sun protection and sunbathing habits among these individuals because they are at higher risk for skin cancer due to their skin cancer surveillance habits, and are therefore an appropriate target for intervention to improve sun protection.

To select correlates for the current study, we integrated constructs from two conceptual models, the Preventive Health Model (PHM) [17,18] and the Theory of Planned Behavior (TPB) [19]. We also based our selection on findings from prior research on correlates of sun protection and sunbathing behaviors from studies of individuals at average risk for skin cancer [20-25]. From the TPB, we included the role of normative influences and considered them as part of broader social influence factors to be examined. Drawing from the PHM, we examined the degree to which background demographic and medical factors (including medical factors of both the FDR and the family member with melanoma), psychological factors, and social influence factors were associated with sun protection and sunbathing. Specific psychological factors we examined included sun protection benefits, sunscreen barriers, benefits of sunbathing, sunscreen self-efficacy, photo-aging concerns, perceived risk and severity of melanoma, and distress about melanoma. The social influence factors we examined included physician recommendation for sun protection, image norms for tanness (i.e., image norms for what is portrayed as attractive in the media), sun protection norms, and sunbathing norms. In addition, we examined whether knowledge variables (i.e., knowledge of sun-protection guidelines and knowledge about sunscreen and sun exposure) were associated with sun protection or sunbathing practices. Few previous studies have examined the association between knowledge and skin cancer prevention behaviors and results have been equivocal [13,26].

The current study had two aims. The first aim was to evaluate demographic, medical, psychological, knowledge, and social influence correlates of sun protection and sunbathing practices among FDRs of melanoma patients. The second, exploratory aim was to examine whether there were unique correlates of sun protection and sunbathing practices. Specifically, we hypothesized that greater perceived sun protection benefits, sunscreen self-efficacy, photo-aging concerns, physician recommendation for sun protection, and sun protection norms would be associated with higher sun protection. In contrast, we hypothesized that greater perceived benefits of sunbathing, lower photo-aging concerns, greater image norms for tanness, and greater sunbathing norms would be associated with higher levels of sunbathing.

## Methods

### Participants and Approach

Data for this study were drawn from the pre-intervention data of a randomized clinical trial evaluating the efficacy of two behavioral interventions to improve skin cancer surveillance and prevention among family members of patients with melanoma [27]. Participants were FDRs of patients recruited from the cutaneous oncology practices at three participating medical centers (Fox Chase Cancer Center, Moffitt Cancer Center, and the University of Pennsylvania Health Systems). Prospective participants were identified from tumor registries or medical records. IRB approval was received for each site. Physicians of record gave permission for their patients to be contacted. Sample recruitment began in February 2006 and ended in June 2009. Eligibility criteria for patients whose FDRs are the focus of this study included: a) newly diagnosed with cutaneous malignant melanoma (CMM) since 2001 but more than 3 months prior to being approached; b) seen at one of the three participating sites; c) greater than 18 years of age; d) English speaking; e) able to give meaningful informed consent; f) does not have a FDR with CMM (to exclude patients with familial melanoma syndrome). Patients who met these criteria were mailed a letter describing the study and subsequently contacted by telephone to determine eligibility. At this time, patients gave permission to contact all of their FDRs and for medical information to be obtained from their medical charts. The Institutional Review Boards at the three participating sites approved this study.

Next, identified FDRs were mailed a letter describing the study. They were contacted by telephone and eligibility was determined. Eligibility criteria for FDRs were: a) at least 20 years of age; b) had not had a total cutaneous examination in the past three years, had done a skin self-examination three or fewer times in the past year, and had a sun protection habits mean score less than four out of five; c) one or more of the following additional risk factors: blonde or red hair, marked freckling on the upper back, history of three or more blistering sunburns prior to age 20, three or more years of an outdoor summer job as a teenager, or actinic keratosis (a precancerous skin condition); d) able to give meaningful informed consent; e) English speaking; f) has residential phone service; g) no personal history of CMM or non-melanoma skin cancer; h) no personal history of dysplastic nevi (abnormal moles); i) only one FDR with CMM. After written informed consent and HIPAA acknowledgement, a baseline telephone survey (Additional file 1) was completed.

Of the 3603 patients approached, 10.3% were ineligible (*n *= 370), 25.6% could not be located (*n *= 923), 35.6% refused (*n *= 1282), and 28.5% of patients provided permission to contact their relatives (*n *= 1028). These 1028 patients provided 3013 FDR names (2.95 per patient). Of these 3013, 43.9% were ineligible (*n *= 1324). Eight hundred fifty-five FDRs were ineligible because they did not meet sun or skin protection criteria, 419 were ineligible because of skin cancer medical history, and 50 were ineligible due to additional risk factors or being under the age of 20 years. Twenty percent could not be located (*n *= 603). Of the 1086 eligible and locatable FDRs identified, 541 refused (49.8%) and 545 (50.2%) enrolled.

A comparison between the 541 FDRs who refused the study with the 545 FDR participants on available demographic information indicated that participants were significantly older than refusers (*t *(759) = 11.5, *p *< .001; *M *_participants _= 46.3, *SD *= 13.3, *M *_refusers _= 31.2, *SD *= 28.7) and that participants were more likely to be female (Percentage female _participants _= 62.4%; Percentage female _refusers _= 46.5%; χ^2 ^(1, 1085) = 27.7, *p *< .001). Participants were also significantly more likely to be offspring of patients (56.1%) than refusers (31%).

### Materials

For each of the multi-item scales assessing psychological factors and social influence factors, a scale score was created by averaging responses across the respective items. Additional information regarding the multi-item scales and internal consistency for these scales are shown in Table 1, and all survey items are contained in an online appendix (Additional file 1).

**Table 1:** Internal Reliability, Sample Items, and Response Options for Multi-Item Scales

Scale	Number of items	α	Sample Item	Response Options
Psychological Factors				
Sun protection benefits	8	.84	If people protected themselves from the sun, they wouldn't be as likely to get skin cancer	1 = *strongly disagree *to 6 = *strongly agree*
Sunscreen barriers	7	.85	The nuisance of applying sunscreen may keep me from using it	1 = *strongly disagree *to 6 = *strongly agree*
Sun protection behavior benefits	6	.69	How much do you think it helps to wear a hat to protect yourself from the harmful rays of the sun?	1 = *not at all helpful *to 5 = *extremely helpful*
Benefits of sunbathing	7	.94	I feel more attractive with a tan	1 = *strongly disagree *to 6 = *strongly agree*
Sunscreen self-efficacy	8	.88	How confident are you that you can use sunscreen while doing outdoor activities in the winter?	1 = *not at all confident *to 5 = *extremely confident*
Photo-aging concerns	3	.84	If I were not to use sun protection, my skin would be very susceptible to wrinkling	1 = *strongly disagree *to 6 = *strongly agree*
Comparative perceived risk	3	.67	How would you rate your chances of developing melanoma compared with other people with a similar family history of melanoma?	1 = *much lower *to 5 = *much higher*
Perceived severity of melanoma	6	.81	How severely would developing melanoma disrupt your personal health and physical comfort?	1 = *not at all disruptive *to 6 = *extremely disruptive*
Social Influence Factors				
Image norms for tanness	5	.62	I think to be a successful TV star, you should have a suntan	1 = *strongly disagree *to 6 = *strongly agree*
Sun protection norms	7	.76	My friends and family wear protective clothing, like a shirt or hat, on a regular basis when in the sun	1 = *strongly disagree *to 6 = *strongly agree*
Sunbathing norms	5	.74	My friends and family sunbathe on a regular basis	1 = *strongly disagree *to 6 = *strongly agree*

#### Demographics

Participants reported their age, sex, race/ethnicity, level of education, marital status, and their relation to the patient with melanoma (i.e., sibling, parent, or offspring).

#### Medical factors

Participants indicated whether they had any form of health insurance, if they had visited a dentist in the past year, and the number of times they had visited a doctor in the past year. Questions also asked about five risk factors for melanoma (e.g., having blonde or red hair as a teenager, having three or more blistering sunburns before the age of 20); we created a total risk factor score by summing across the five items. For each melanoma patient, the disease stage at diagnosis and length of time since diagnosis was abstracted from medical records.

#### Psychological factors

The measures of sun protection benefits and sunscreen barriers were taken from Jackson and Aiken [21]. Sun protection behavior benefits were assessed using a measure developed by Glanz and colleagues [28]. Measures of benefits of sunbathing, sunscreen self-efficacy (for which the items asked about confidence in using sunscreen in various situations), and photo-aging concerns were taken from Jackson and Aiken [29]. Four items assessed perceived risk of developing melanoma [13]. One of the four items asked participants to indicate their overall perceived risk of developing melanoma during their lifetime (rated from 0 = *not at all likely *to 100 = *extremely likely*). The remaining three items asked about different aspects of comparative perceived risk. Perceived severity of melanoma was assessed using a measure adapted from Aiken and colleagues [30]. Distress about melanoma was assessed with a single item ("How distressed are you currently about the diagnosis and treatment of your family member's melanoma"?) with response options from 1 = *not at all distressed *to 5 = *extremely distressed*.

#### Knowledge

Two multiple-choice items asked about knowledge of sun protection guidelines (i.e., the recommended minimum level of sunscreen sun protection factor (SPF) to use when in the sun, and the recommended hours during the day when people are advised to limit sun exposure). We summed the number of correct responses to the two items. Knowledge about sunscreen and sun exposure was assessed with 11 true-false items drawn from previous research (e.g., "To work best, sunscreen needs to be applied a half-hour before you go outside") [13]. We summed the number of correct responses to the 11 items.

#### Social influence factors

Three items drawn from Manne et al. [13] assessed physician recommendations for sun protection. The items asked whether a doctor had ever told the participant to reduce the amount of time spent in the sun, wear a hat or long sleeves when in the sun, or to use sunscreen regularly. Responses were summed across the three items. Measures of image norms for tanness (i.e., attitudes about tanness and paleness among celebrities), sun protection norms (i.e., sun protection practices and attitudes among friends and family), and sunbathing norms (i.e., sunbathing practices and attitudes among friends and family) were drawn from prior research [13,29].

#### Outcome variables: Sun protection behaviors and sunbathing

Sun protection behaviors were measured using a 5-item measure that asked about the frequency (from 1 = never to 5 = always) of engaging in the following behaviors when out in the sun for more than 30 minutes: using a sunscreen with an SPF of 15 or more, wearing a hat, wearing a shirt with long sleeves, staying in the shade, and wearing sunglasses [28]. Responses were averaged across the five items. Sunbathing was assessed with a single item that asked about the frequency (from 1 = *never *to 5 = *always*) of spending time in the sun to get a tan last summer.

### Statistical Analyses

All statistical analyses were conducted using SAS (version 9.2), and a cutoff of *p *< .05 was used to determine statistical significance. The primary analyses consisted of a series of multiple regressions to examine correlates of the two outcomes, sun protection behaviors and sunbathing. In order to account for the fact that some participants were members of the same family, all of the regression analyses were conducted using a generalized estimating equations (GEE) approach (PROC GENMOD in SAS), with the assumption of an exchangeable correlation matrix. Regression models for the sun protection behaviors measure were fit under the assumption of a normal distribution. Data from the sunbathing measure were positively skewed, and thus all regression models for that outcome were fit under the assumption of a gamma distribution. The *p *values reported for the regression analyses are from type 3 tests of model effects. We used the following analytic approach with sun protection behaviors and sunbathing as separate outcome variables in a series of GEE regression analyses. First, separately for each category of potential correlates (i.e., demographics, medical factors, psychological factors, knowledge, and social influence factors), we included all of the variables in that category as independent variables in a single regression model. Next, across all of the categories, the independent variables that were significantly associated with the outcome in the initial analyses were included together in a final regression model. There was no evidence of multicollinearity for any of the regression models.

## Results

### Descriptive Statistics

There were few missing data, with no more than eight individuals missing data for any one variable. Table 2 shows descriptive statistics for all of the study variables. With regard to the outcome variables, there was a small-to-moderate inverse correlation (*r*_s _= -.20, *p *< .001) between sun protection behaviors and sunbathing. Average levels of sun protection behaviors were close to the middle of the 5-point scale (*M *= 2.8, where 3 = *sometimes*), whereas average levels of sunbathing were relatively low (*M *= 1.9, where 2 = *rarely*).

**Table 2:** Descriptive Statistics for the Study Variables

Variable	%	*M*	*SD*	Range
*Demographics*				
Age		46.3	13.3	20-85
Sex				
Female	62.4			
Male	37.6			
Ethnicity				
White	99.1			
Non-white	.0.9			
Education level				
High school or less	15.1			
Some college	28.6			
College degree	31.4			
At least some graduate school	25.0			
Marital status				
Married	68.1			
Not married	31.9			
Relation to patient with melanoma				
Offspring	56.1			
Sibling	31.7			
Parent	11.9			
*Medical Factors*				
Have health insurance	94.1			
Visited a dentist in the past year	80.7			
Doctor visits in the past year				
0	7.2			
1-2	33.9			
3-4	26.8			
≥ 5	32.1			
Number of melanoma risk factors		2.6	1.1	1-5
Patient disease stage				
0	17.1			
1	40.2			
2	22.9			
3	13.9			
4	5.9			
Years since patient's diagnosis		2.7	1.5	0.2-6.2
< 1	13.1			
1 to < 2	27.9			
2 to < 3	16.3			
3 to < 4	20.9			
*Psychological Factors*				
Sun protection benefits		4.6	0.8	2.1-6.0
Sunscreen barriers		2.3	1.0	1.0-5.4
Sun protection behavior benefits		4.3	0.5	2-5
Benefits of sunbathing		3.7	1.2	1-6
Sunscreen self-efficacy		2.8	1.0	1-6
Photo-aging concerns		5.1	1.0	1-6
Overall perceived risk		46.9	22.7	0-100
Comparative perceived risk		3.5	0.6	1-5
Perceived melanoma severity		4.4	0.8	1-6
Distress about patient's melanoma		2.8	1.2	1-5
*Knowledge*				
Knowledge of sun protection guidelines		0.9	0.7	0-2
Knowledge about sunscreen and sun exposure		9.1	1.5	3-11
*Social Influence Factors*				
Physician recommendation for sun protection		0.6	0.8	0-3
Image norms for tanness		3.5	0.8	1.2-6.0
Sun protection norms		3.5	0.8	1.1-5.4
Sunbathing norms		3.7	1.0	1.0-6.0
*Outcome Variables*				
Sun protection behaviors		2.8	0.7	1.0-3.8
Sunbathing		1.9	0.1	1-5

*Note: N *= 545

### Correlates of Sun Protection Behaviors

Among the demographic factors, education was positively associated with sun protection behaviors (parameter estimate [*b*] = 0.05, *SE *= 0.03, *p *= .049). The relation of the participant to the patient with melanoma was also significantly associated with sun protection behaviors (*p *= .041), such that siblings (*b *= -0.16, *SE *= 0.07) and parents (*b *= -0.25, *SE *= 0.11) engaged in fewer sun protection behaviors than did offspring of patients. None of the medical factors were significantly associated with sun protection behaviors (*p*s ≥ .086). For the psychological factors, higher sun protection behaviors were found among individuals reporting fewer benefits of sunbathing (*b *= -0.08, *SE *= 0.02, *p *= .001), greater sunscreen self-efficacy (*b *= 0.15, *SE *= 0.03, *p *< .001), and greater photo-aging concerns (*b *= 0.07, *SE *= 0.03, *p *= .039). With regard to the knowledge variables, individuals with greater knowledge about sunscreen and sun exposure (*b *= 0.04, *SE *= 0.02, *p *= .024) had higher sun protection behaviors. Knowledge about sun protection guidelines (*p *= .153) was not associated with sun protection behaviors. Among the social influence factors, individuals reporting greater sun protection norms (*b *= 0.14, *SE *= 0.04, *p *< .001) or lower sunbathing norms (*b *= -0.07, *SE *= 0.03, *p *= .012) had higher sun protection behaviors.

A final regression model was tested in which all of the significant correlates from the preceding analyses were included as independent variables, with sun protection behaviors as the dependent variable. As shown in Table 3, the relation of the participant to the melanoma patient, knowledge about sunscreen and sun exposure, and sunbathing norms were not significantly associated with sun protection behaviors. Higher sun protection behaviors were found among individuals with more education, individuals reporting fewer benefits of sunbathing, greater sunscreen self-efficacy, greater photo-aging concerns, and greater sun protection norms.

**Table 3:** Results of Generalized Estimating Equations (GEE) Multiple Regression Analysis Examining Correlates of Sun Protection Behaviors

Variable	Parameter Estimatea	95% CI	*p *value^b^
Education	0.06	0.00, 0.11	.034
Relation to patient with melanoma			.326
Offspring	Ref		
Sibling	-0.07	-0.19, 0.05	
Parent	-0.10	-0.26, 0.05	
Benefits of sunbathing	-0.09	-0.14, -0.04	< .001
Sunscreen self-efficacy	0.13	0.07, 0.19	< .001
Knowledge about sunscreen and sun exposure	0.02	-0.02, 0.06	.291
Sun protection norms	0.10	0.03, 0.18	.005
Sunbathing norms	-0.02	-0.08, 0.04	.440

*Note*: ^a ^Parameter estimates are unstandardized regression coefficients. ^b ^*p *values are from type 3 tests of model effects.

### Correlates of Sunbathing

Of the demographic factors examined, age (*b *= -0.01, *SE *= 0.002, *p *< .001) and sex (*b *= 0.16, *SE *= 0.05, *p *= .002) were significantly associated with sunbathing, with younger individuals and women reporting more sunbathing. The only medical factor that was significantly associated with sunbathing was visiting a dentist in the past year (*b *= 0.15, *SE *= 0.07, *p *= .039). In the analysis examining the association between the psychological factors and sunbathing, more frequent sunbathing was reported by those reporting fewer sunscreen barriers (*b *= -0.10, *SE *= 0.02, *p *< .001) and those reporting greater benefits of sunbathing (*b *= 0.22, *SE *= 0.02, *p *< .001). Neither of the knowledge variables was significantly associated with sunbathing (*p*s ≥ .180). Of the social influence factors examined, more frequent sunbathing was found among individuals with lower endorsement of image norms for tanness (*b *= -0.10, *SE *= 0.03, *p *< .001) and those with higher sunbathing norms (*b *= 0.28, *SE *= 0.02, *p *< .001). Neither physician recommendations for sun protection nor sun protection norms were significantly associated with sunbathing (*p*s ≥ .157).

All of the significant correlates from the preceding analyses were included as independent variables in a final model with sunbathing as the outcome variable. As shown in Table 4, with the exception of visiting a dentist in the past year, each correlate in the model was significantly associated with sunbathing. More frequent sunbathing was found among younger individuals, women, those reporting fewer sunscreen barriers, individuals reporting greater benefits of sunbathing, and those with lower endorsement of image norms for tanness or higher sunbathing norms.

**Table 4:** Results of Generalized Estimating Equations (GEE) Multiple Regression Analysis Examining Correlates of Sunbathing

Variable	Parameter Estimate^a^	95% CI	*p *value^b^
Age	-0.01	-0.01, -0.01	< .001
Sex	0.13	0.05, 0.21	.002
Visited a dentist in the past year	0.06	-0.05, 0.16	.269
Sunscreen barriers	-0.05	-0.09, -0.02	.006
Benefits of sunbathing	0.17	0.14, 0.21	< .001
Image norms for tanness	-0.13	-0.18, -0.09	< .001
Sunbathing norms	0.19	0.15, 0.24	< .001

*Note*: ^a ^Parameter estimates are unstandardized regression coefficients. ^b ^*p *values are from type 3 tests of model effects.

## Discussion

Results indicated that demographic, psychological, and social influence factors contributed to sun protection and sunbathing among close family members who are not compliant with sun protection or other skin surveillance practices. Relatives who reported higher sun protection practices were more educated, endorsed fewer benefits of sunbathing, greater sunscreen self-efficacy, had greater concerns about the effects of UV on photo-aging, and greater perceptions of sun protection norms. FDRs who reported more sunbathing were younger, more likely to be female, endorsed fewer barriers to using sunscreen, perceived more benefits of sunbathing, lower image norms for tanness, and endorsed higher sunbathing norms. Several medical, psychological, knowledge, and social factors were not associated with either sun protection or sunbathing. Overall, findings were consistent with previous literature as well as with the conceptual framework guiding this work. The results were relatively consistent with our exploratory hypotheses regarding the unique factors associated with sun protection or sunbathing. It is interesting to note that, although we selected our participants based upon low levels of sun protection and skin surveillance behaviors, the levels of sunbathing in our sample were relatively low and comparatively lower than rates of sunbathing [11,16] and sunburn [15,16] reported in previous studies. In the discussion that follows, we consider how the results of the current study extend what is known about correlates of sun protection and sunbathing among family members, and we also address clinical and research implications of the findings.

Given that the study focused on close relatives of individuals with melanoma, it is noteworthy that characteristics of the patient's disease, such as stage and time since diagnosis as well as attitudinal variables typically associated with the severity of cancer such as distress about the proband's melanoma, disease severity, and perceived risk, were not associated with sun protection or sunbathing. The fact that disease characteristics were not associated with sun protection is consistent with our previous study of family members of melanoma patients [13] as well as prior work with family members of colorectal cancer patients [31]. With regard to disease severity, perceived risk, and distress about the proband's cancer, our results are also consistent with our previous research [13]. These findings suggest that family members may not be influenced to alter sun protection or exposure by the severity of the patient's cancer or their own melanoma risk. However, it is possible that the lack of association between all of these factors and relatives' behavior is due to the fact that they were not aware of important facts about melanoma because the proband and relative did not have an in-depth discussion about this topic. During this discussion, it is likely the proband would discuss the cancer in more detail in terms of the level of risk conferred upon the family member. Family communication has been linked with engagement in cancer screening practices among family members at increased cancer risk (e.g., [32-34]). For similar reasons, it is also possible that the closeness of the relationship with the proband would have had a stronger association with sun protection and sunbathing practices than severity, risk, and distress about the proband's cancer, as this variable has been associated with other types of cancer risk reduction behavior [30,35]. Unfortunately, this measure was not included in this study. Without a qualitative examination of each family's communication about melanoma risk, it is difficult to conclude why these variables were not associated with sun protection and sunbathing practices.

Consistent with previous research older age was associated with less sunbathing [23,26]. It is interesting that physician recommendation for sun protection was not associated with sun protection or sunbathing, which is not consistent with previous work evaluating correlates of sun protection among family members of patients with melanoma [13]. It is possible that this population of family members had not had contact with a dermatologist and thus there was less opportunity for a dermatologist to influence the adoption of sun protection practices. The other social influence factors we examined were varying types of norms. Sun protection norms were associated with sun protection and sunbathing norms were associated with sunbathing behavior. These findings suggest that peers' attitudes and behaviors may be more important than expert recommendations. Consistent with our expectations, sunscreen self-efficacy was associated with sun protection but not sunbathing.

In line with previous research, greater perceived benefits of sunbathing and higher perceptions that family and friends engage in tanning behaviors were associated with greater sunbathing [29]. However, a greater endorsement of positive image norms for tanness was associated with a lower frequency of sunbathing, which is opposite to the effect found in prior research [29]. One factor that might account for these discrepant findings is that our sample included both men and women and was older and at higher risk for skin cancer than the mostly female, college-aged samples studied previously. It is possible that perceptions of societal standards of attractiveness are more influential in personal choices among younger women as compared with older samples comprised of both genders, as well as among individuals at increased risk for melanoma. In addition, future studies should attempt to distinguish the role of perceptions of societal values versus the role of agreement with those values. The present measure did not separate perceptions of values from endorsement of them. Participants who reported having fewer barriers to using sunscreen engaged in more sunbathing. It is possible that individuals who sunbathe are generally more likely to use sunscreen because they are going to tan and thus they report fewer barriers to using it [36-39]. This may also be more likely to be the case among middle-aged and older individuals than among college women.

### Study Strengths and Limitations

The strengths of this study include the large sample size, the focus on family members, the focus on high risk individuals who did not engage in regular sun protection and skin surveillance, the inclusion of sunbathing as an outcome, and the inclusion of previously unstudied correlates of behavior such as the medical status of the affected family member and the level of psychological distress about the affected family member's cancer. This study is also one of few to focus on an older sample of men and women.

There are several study limitations. The cross-sectional methodology precludes the ability to infer causal relationships. The sample was comprised of relatively well-educated and married individuals, and almost half the sample was comprised of patients' offspring. Female and older relatives were more likely to participate. It is not known whether levels and correlates of sun protection and sunbathing would have differed with a more heterogeneous sample. It is also not known whether the patients who provided family member names differed from those patients who we were not able to contact or who declined to provide family member names.

### Implications

This study extends what is known about sun protection and sunbathing from previous work conducted on average risk populations to a population of high risk individuals. Although caution should be used in using cross-sectional results to guide interventions, these results provide information regarding the factors that might be focused on in future interventions to address sun protection and sunbathing in this population. In terms of implications for interventions to improve sun protection for at-risk family members, self-efficacy for using sunscreen could be highlighted by discussing recent developments in sunscreen manufacturing and marketing. These include the fact that SPF 15 or higher has been incorporated into many daily-use skin products such as moisturizers and that sunscreens can be sprayed on and can be purchased in unscented, non-greasy versions. Because our data suggest that men are more likely to consider sunscreen a hassle and a nuisance and not endorse the preventive influence sunscreen has on cosmetic effects of aging (unpublished data), future studies may need to employ qualitative methods to identify strategies for increasing positive perceptions of sunscreen. Emphasizing detrimental cosmetic and photo-aging effects of sun exposure through appearance-based materials, such as age-progressed pictures of the family member, may also prove beneficial. Overall, interventions to reduce sunbathing among FDRs of patients with melanoma should attempt to counteract both perceived benefits of sunbathing and normative influences of family and friends to sunbathe. Emphasis should also be placed on reasons why sunbathing should be avoided (e.g., sunscreen is not 100% effective) and should target younger family members by emphasizing the aging effects of sunbathing on the skin. In view of the evidence indicating that the correlates of sun protection and sunbathing are not the same, interventions may be more effective if they include separate components to address sun protection and sunbathing behaviors. Finally, because health care professionals did not influence sun protection and sunbathing, general practitioners should ask about a family history of skin cancer and refer these individuals to a dermatologist. In view of the rising incidence of melanoma, the development and testing of such interventions is an important public health issue.

In terms of recommendations for future research, we found it more difficult to recruit younger and male relatives into the study. Recruitment materials and more intensive recruitment efforts targeted towards younger relatives and men as well as educating melanoma probands about ways to facilitate participation of their younger and male relatives into the study may facilitate a higher uptake in this population of probands. Previous research has suggested that individuals with a family history of melanoma are more likely to speak to their female relatives about melanoma [32] and therefore it is possible that a greater proportion of male and younger relatives will participate in future research if family communication to male relatives is fostered.

## Conclusions

Demographic, psychological, and social influence factors contributed to sun protection and sunbathing practices among melanoma patients' close family members who were not compliant with sun protection or other skin surveillance practices. Less educated and female relatives are less compliant with recommended practices and may benefit from targeted interventions to improve their sun protection and sun exposure practices. Attitudinal factors such as concerns about photo-aging and the perceived benefits of sunbathing were key, and the sun protection and tanning practices of family, friends, and celebrities also played a role. Additionally, attitudes toward sunscreen use including self-efficacy and perceived barriers contributed to skin protection and sunbathing practices, respectively. These findings suggest that the effectiveness of behavioral interventions to improve these practices may be improved if we target less educated and female relatives as well as the attitudes and social influences that contribute to low levels of sun protection and sun avoidance in this population of at-risk family members.

## Competing interests

The authors declare that they have no competing interests.

## Authors' contributions

SLM conceived the study, coordinated data collection, assisted in data analyses, and was the primary author of the manuscript. EJC designed the study, conducted data analyses, and assisted in writing the manuscript. PBJ participated in the design of the study, coordinated data collection, and assisted with writing the manuscript. MM coordinated data collection and assisted with writing the manuscript. CJH assisted with the data interpretation and assisted in writing the manuscript. SL participated in the initial design of the study and coordinated data collection. All authors read and approved the final manuscript.

## Pre-publication history

The pre-publication history for this paper can be accessed here:

http://www.biomedcentral.com/1471-2458/11/122/prepub

## Supplementary Material

Additional file 1 **Study Survey Items**. The file contains all of the survey items used in the study. Click here for file
