# Supplementary material for: Sun protection and sunbathing practices among at-risk family members of patients with melanoma
Source: BMC Public Health. 2011 Feb 21;11:122. doi: 10.1186/1471-2458-11-122 (PMC3050750; doi:10.1186/1471-2458-11-122)
Supplement: Additional file 1 — Study Survey Items. The file contains all of the survey items used in the study. [file 1471-2458-11-122-S1.DOC]

**DEMOGRAPHICS**

1. What is your gender? Male ___

Female ___

1. When is your birth date? ______________________

3. What is your ethnic identification?

White (Caucasian) 1

Black (African American) 2

Asian/ Pacific Islander 3

Hispanic 4

American Indian/Native American 5

Other (please specify) _________________

4. How much schooling have you completed?

0 to 4 1

Grades 5-8 2

Some high school 3

Finished high school 4

1-3 years of college 5

Trade or business school 6

4 years of college 7

Some graduate school 8

Graduate degree 9

5. What is your present marital status?

Single, never married 1

Married/Partnered 2

Separated 3

Divorced 4

Widowed 5

6. Participant’s relation to proband: Sibling

Parent

Offspring

**MEDICAL FACTORS**

1. Are you currently covered by major medical/health insurance?

- Yes
- No

2. Do you have regular dental check-ups (at least once a year)?

- Yes
- No

3. How many times have you seen a doctor (of any kind) in the past year?

None___ 1-2 times ____ 3-4 times ____ 5 or more times ____

**OBJECTIVE RISK FACTORS**

1. Had blonde or red hair as a teenager: (yes)

2. Has “**EXTENSIVE”** or “**MODERATE FRECKLING**” on their upper back: (yes to either)

3. Had **THREE** or more blistering sunburns before the age of 20: (yes)

4. Worked outdoors for the summer months for **THREE** or more years before the age of 20: (yes)

5. “**FREQUENTLY”** spent summers outdoors as a child or teenager: (yes)

# **PSYCHOLOGICAL FACTORS**

**Sun Protection Benefits (items 1, 2, 3, 4, 5, 13, 14, 15) and Barriers (items 6, 7, 8, 9, 10, 11, 12)**

Instructions: Please read the following statements and use the scale below to indicate how much you agree with each one.

1 = Strongly Disagree

2 = Moderately Disagree

3 = Somewhat Disagree

4 = Somewhat Agree

5 = Moderately Agree

6 = Strongly Agree

_____1. Wearing sunscreen with an SPF of at least 15 when I am in the sun would reduce my chances of getting skin cancer.

_____2. Whether or not a person develops skin cancer is related to how frequently they use sunscreen while spending time in the sun.

_____3. If people protected themselves from the sun, they wouldn’t be as likely to get skin cancer.

_____4. If more people used sunscreen with an SPF of at least 15 regularly, there would be fewer cases of skin cancer.

_____5. Whether or not a person develops age spots and wrinkles is related to how frequently they use sunscreen while spending time in the sun.

_____6. The cost of sunscreen may possibly keep me from using it.

_____7. The hassle of having to carry sunscreen with me may keep me from using it.

_____8. Remembering to apply sunscreen may pose as a problem for me and may possibly keep me from using it.

_____9. The unpleasant smell of sunscreen might keep me from using it.

_____10. The nuisance of applying sunscreen may keep me from using it.

_____11. The unpleasant sensation caused by sunscreen may keep me from using it.

_____12. The pain of having to reapply sunscreen might keep me from using it.

_____13. If more people used sunscreen with an SPF of at least 15 regularly, people would look younger longer.

_____14. If people protected themselves from the sun, they wouldn’t age so fast.

_____15. Wearing sunscreen with an SPF of at least 15 regularly when I am in the sun will reduce my chances of getting age spots and wrinkles.

**Sun Protection Behavior Benefits**

Instructions: Please use the scale below to rate how much you think it helps to do each of the following to protect yourself from the harmful rays of the sun.

1 = Not at all

2 = A little helpful

3 = Somewhat helpful

4 = Very helpful

5 = Extremely helpful

____1. Use sunscreen.

____2. Wear shirt with sleeves.

____3. Wear a hat.

____4. Wear sunglasses.

____5. Have a good base suntan.

____6. Stay in shade or under umbrella.

____7. Limit the hours outdoors between 10 a.m. – 4 p.m.

**Benefits of Sunbathing**

Instructions: Please use the scale below to indicate how much you agree with each of the following statements.

1 = Strongly Disagree

2 = Moderately Disagree

3 = Somewhat Disagree

4 = Somewhat Agree

5 = Moderately Agree

6 = Strongly Agree

____1. I feel more attractive with a tan.

____2. A sun-tanned person looks healthier.

____3. Having a tan makes my skin look better.

____4. A sun-tanned person looks more attractive.

____5. A tan makes me feel better about myself.

____6. I feel healthier with a tan.

____7. I have more self-confidence with a tan.

**Sunscreen Self-Efficacy**

Instructions: Please use the scale below to rate how confident you are that you could really do the things I will state next, whether or not you presently use sunscreen with a SPF of 15 or higher.

1 = Not at all confident

2 = Somewhat confident

3 = Moderately confident

4 = Very confident

5 = Extremely confident

______1. How confident are you that you can use sunscreen while in the sun even if you

are not going to be out long?

______2. How confident are you that you can use sunscreen on the exposed parts of

your body (not just your face) when you are in the sun?

______3. How confident are you that you could use sunscreen while doing outdoor

activities in the winter?

______4. How confident are you that you can use sunscreen on every part of your body

that is not covered by clothing?

______5. How confident are you that you can use sunscreen everyday, even when you

are not planning on spending time in the sun?

______6. How confident are you that you can make using sunscreen a part of your daily

routine like brushing your teeth?

______7. How confident are you that you can use sunscreen even when you are feeling

too lazy to bother to use it?

______8. How confident are you that you can use sunscreen while doing outdoor

activities?

**Photo-Aging Concerns**

Instructions: Please use the scale below to indicate how much you agree with each of the following statements.

1 = Strongly disagree

2 = Moderately disagree

3 = Somewhat disagree

4 = Somewhat agree

5 = Moderately agree

6 = Strongly agree

_____1. If I were not to use sun protection, I would be very susceptible to getting age

spots.

_____2. If I were not to use sun protection, my skin would be very susceptible to

wrinkling.

_____3. If I were not to use sun protection, my skin would be very susceptible to sun

damage.

**Overall Perceived Melanoma Risk**

1. On a scale from 0 (not at all likely) to 100 (extremely likely) how likely do you think it is that you will develop melanoma in your lifetime? _________

**Comparative Perceived Melanoma Risk**

1. How would you rate your chances of developing MELANOMA as compared with the average person your own age? Do you think your chances are: (CHOOSE ONE)

- 1. Much higher than other people
  2. A little higher than other people
  3. The same as other people
  4. A little lower than other people
  5. Much lower than other people

2. How would you rate your chances of developing MELANOMA as compared with other people with a similar family history of MELANOMA? Do you think your chances are: (CHOOSE ONE)

1. Much higher than other people
2. A little higher than other people
3. The same as other people
4. A little lower than other people
5. Much lower than other people

3. How has the diagnosis of MELANOMA in your relative(s) affected your own chances for this illness? Are your chances now: (CHOOSE ONE)

a. Much higher

b. A little higher

c. The same

d. A little lower

e. Much lower

**Perceived Severity of Melanoma**

Instructions: Please answer the following questions by choosing the number that is closest to your answer.

1. How severely would developing melanoma disrupt each of the following aspects of your life?

Not at all Extremely

Disruptive Disruptive

a. Personal health and physical comfort 1 2 3 4 5 6

b. Emotional well-being 1 2 3 4 5 6

c. Physical attractiveness and appearance 1 2 3 4 5 6

2. Overall, how disruptive would melanoma cancer be in your life?

Not at all Extremely

Disruptive Disruptive

1 2 3 4 5 6

3. How severe would the health consequences of melanoma cancer be for you if it was caught *early*?

Not at all Extremely

Severe Severe

1 2 3 4 5 6

4. How severe would the health consequences of melanoma cancer be for you if it was caught *late*?

Not at all Extremely

Severe Severe

1 2 3 4 5 6

**Distress About Melanoma**

# 1. How distressed are you currently about the diagnosis and treatment of your family member’s

# melanoma?

# 1 2 3 4 5

# Not at all Somewhat Extremely

# distressed distressed distressed

**KNOWLEDGE**

**Knowledge of Sun Protection Guidelines**

1. How strong of a sun protection factor do the American Cancer Society and the American Academy of Dermatology suggest people use when spending time in the sun?
   1. A minimum of SPF 8
   2. A minimum of SPF 10
   3. A minimum of SPF 15 **[correct answer]**
   4. A minimum of SPF 30
   5. I don’t know
2. During which hours do the American Cancer Society and the American Academy of Dermatology

strongly suggest people should avoid sun exposure?

- 1. between 9 AM and 12 PM
  2. between 10 AM and 4 PM **[correct answer]**
  3. between 12 PM and 3 PM
  4. between 2 PM and 6 PM
  5. I’m not sure

**Knowledge About Sunscreen and Sun Exposure**

Instructions: Please answer each question by answering True or False. **[NOTE: correct answers (true or false) are denoted by bold text.]**

**True** False 1. To work best, sunscreen needs to be applied a half-hour before you go outside.

True **False** 2. If a person wears a sun protection factor in sunscreen of 15 or more, he

or she can stay outside in the sun for more than three hours.

True **False** 3. There is no need to worry about getting skin cancer if you are exposed

to the sun ten to twenty minutes.

True **False** 4. If a person is in the sun for only 2 weeks a year, he or she is not likely to get

skin cancer.

**True** False 5. Sun exposure in childhood increases the chances of getting melanoma as an adult.

**True** False 6. Sun exposure is the greatest preventable risk factor for melanoma.

**True** False 7. Sun exposure when you are young increases your risk for developing melanoma.

**True** False 8. You can burn more severely on hazy or windy days; such conditions

accentuate the harmful effects of ultraviolet radiation (“tanning rays”).

**True** False 9. You can still burn on overcast days, and even while sitting in the shade

of an umbrella.

**True** False 10. Wet skin allows more ultraviolet rays to penetrate the outer, protective

layers of skin.

**True** False 11. Children, in particular, should be protected from traumatic sunburns

due to the greatly increased risk of melanoma later in life.

**SOCIAL INFLUENCE FACTORS**

**Physician Recommendations for Sun Protection**

1. Has your doctor (or other health care professional) ever told you to reduce the amount of time you spend in the sun?

- Yes
- No

2. Has your doctor (or other health care professional) ever told you to wear a hat or long sleeves when you are out in the sun?

- Yes
- No

3. Has your doctor (or other health care professional) ever told you to wear sunscreen regularly?

- Yes
- No

**Image Norms for Tanness**

Instructions: Please use the scale below to indicate how you agree or disagree with each of the following statements.

1 = Strongly Disagree

2 = Moderately Disagree

3 = Somewhat Disagree

4 = Somewhat Agree

5 = Moderately Agree

6 = Strongly Agree

____1. People in the media (celebrities, movie stars) always seem to have a suntan.

____2. I believe that there is a trend towards paler models. **[reverse coded]**

____3. I think that to be a successful TV star, you should have a suntan.

____4. It seems that society wants people to be tanned and attractive.

____5. I can think of many TV stars who are pale and attractive. **[reverse coded]**

**Sun Protection Norms**

Instructions: Please use the scale below to indicate how you agree or disagree with each of the following statements.

1 = Strongly Disagree

2 = Moderately Disagree

3 = Somewhat Disagree

4 = Somewhat Agree

5 = Moderately Agree

6 = Strongly Agree

____1. My friends and/or family wear protective clothing, like a shirt or a hat, on a regular basis

when in the sun.

____2. My friends and/or family use sunscreen with an SPF of at least 15 when they are in the

sun.

____3. My friends and/or family go to all lengths to protect their skin from the sun.

____4. My friends and/or family think I should wear sunscreen with an SPF of at least 15 when

I am in the sun.

____5. Most people use sunscreen when they are outdoors.

____6. Most people wear hats when they are outdoors.

____7. Most people cover up when they are outdoors.

**Sunbathing Norms**

Instructions: Please use the scale below to indicate how you agree or disagree with each of the following statements.

1 = Strongly Disagree

2 = Moderately Disagree

3 = Somewhat Disagree

4 = Somewhat Agree

5 = Moderately Agree

6 = Strongly Agree

____1. My friends and/or family sunbathe on a regular basis.

____2. Most of my friends and/or family are tan.

____3. It would be okay with my friends and/or family if I sunbathed.

____4. Most of my friends and/or family feel that a suntan is a good thing.

____5. My friends and/or family disapprove of people who tan. **[reverse coded]**

**OUTCOME VARIABLES**

**Sun Protection Behaviors**

Instructions: Please use the scale below to indicate how you agree or disagree with each of the following statements.

1 = Never

2 = Rarely

3 = Sometimes

4 = Often

5 = Always

____ 1. Do you use a sunscreen with an SPF of at least 15 consistently? That is, whenever you know you will be out in the sun for more than 30 minutes?

____ 2. Do you wear a hat on a regular basis when you are exposed to the sun for more than 30 minutes?

____ 3. Do you consistently wear a shirt with sleeves on a regular basis when exposed to the sun for more than 30 minutes?

____ 4. Do you consistently stay in the shade or under an umbrella when out in the sun for more than 30 minutes?

____ 5. Do you consistently wear sunglasses when exposed to the sun for more than 30 minutes?

**Sunbathing**

1. How often did you spend time in the sun to get a tan last summer?
   1. Never
   2. Rarely
   3. Sometimes
   4. Often
   5. Always
